# 209. Population Pharmacokinetics of Meropenem-Vaborbactam in Acutely Ill Hospitalized Patients with Various Degrees of Renal Dysfunction

**DOI:** 10.1093/ofid/ofae631.067

**Published:** 2025-01-29

**Authors:** Griffin Reed, Adrian Valadez, Erin K McCreary, Ellen G Kline, Brandon Smith, Michael N Neely, Kevin M Squires, Ryan K Shields, Nathaniel J Rhodes

**Affiliations:** Midwestern University, Peoria, AZ; Midwestern University, Peoria, AZ; University of Pittsburgh Medical Center, Pittsburgh, PA; University of Pittsburgh, Pittsburgh, Pennsylvania; University of Pittsburgh Medical Center, Pittsburgh, PA; The Saban Research Institute, Children’s Hospital Los Angeles, University of Southern California, Los Angeles, CA, USA, Los Angeles, California; University of Pittsburgh, Pittsburgh, Pennsylvania; University of Pittsburgh, Pittsburgh, Pennsylvania; Midwestern University, Peoria, AZ

## Abstract

**Background:**

Pharmacokinetic-pharmacodynamic (PK/PD) target attainment rates for meropenem-vaborbactam (MV) in real-world acutely ill patients are poorly defined. We sought to evaluate target attainment rates for MV in patients with various degrees of renal dysfunction including those requiring continuous renal replacement therapy (CRRT).

**Figure 1.**
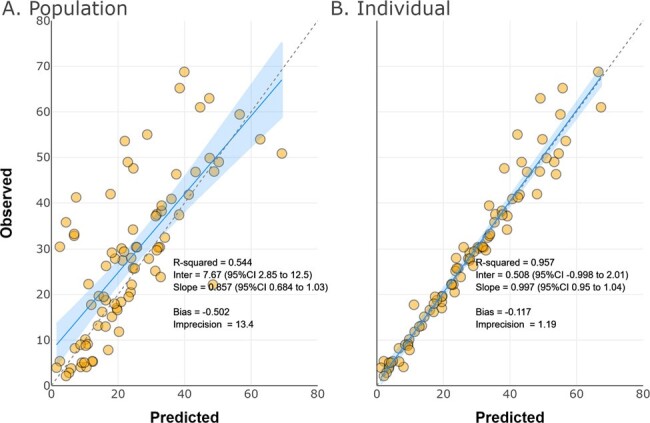
Observed versus predicted meropenem concentrations from the population (A) and individual (B) model fits

**Methods:**

Patients treated with MV from December 2017 to July of 2022 were included. MV was dosed according to institutional protocols based on renal function and indication. After informed consent, opportunistic plasma samples were collected. Plasma MEM and VAB were quantified using a validated LC-MS/MS assay. Patients with acute kidney injury, and those requiring CRRT were included. Patients requiring hemodialysis were excluded. Multiple compartmental models and covariate effects [e.g., Cockcroft Gault calculated creatinine clearance (CRCL) per 1.73m^2^ on clearance (CL) and total body weight (WT) on volume (Vd)] were tested. Analyses were performed using Pmetrics 2.1.1 for R. A free (*f*) fraction of 98% and 67% were assumed for MEM and VAB, respectively. Targets of 40% *f*T _>MIC_ and 100% *f*T _>MIC_ for MEM and *f*AUC:MIC of 38 for VAB were evaluated vs. the MIC_50_ and full EUCAST MIC distribution for *K. pneumoniae* (https://mic.eucast.org/).

**Figure 2.**
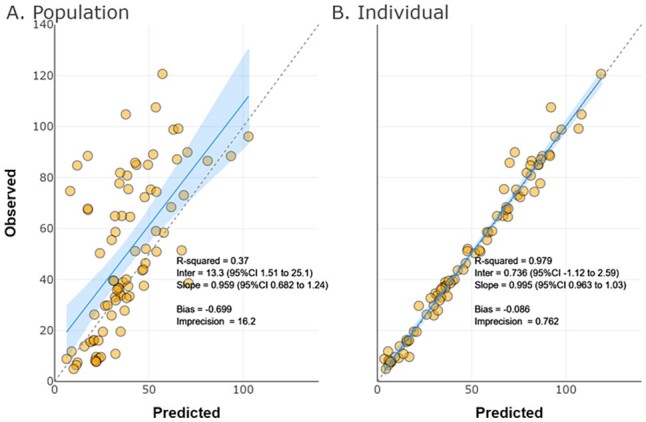
Observed versus predicted vaborbactam concentrations from the population (A) and individual (B) model fits

**Results:**

18 (50% female) patients aged 54±14 years contributed 83 paired MV observations. Baseline CRCL was 114±102 mL/min/1.73m^2^, and baseline WT was 91±31 kg. Three patients received CRRT. A one-compartment, two-input, two-output, population PK model was fit for purpose (MEM Fig1, VAB Fig2). Non-CRRT MEM CL was higher than VAB CL (median 10.6 vs. 6.8 L/hr). CRRT MEM CL was also higher than VAB CL (median 4.1 vs. 2.7 L/hr). However, MEM Vd was similar to VAB Vd (median 23.9 vs. 21.5 L) (Table1). The simulated PTA vs. the EUCAST MIC_50_ of 0.75/8 was high for MEM and VAB targets (Table2). The simulated CFR for MEM was >80% with 4g IV every 8 hr at a 40% *f*T _>MIC_ goal but was reduced at a 100% *f*T _>MIC_ goal whereas the CFR for VAB approached 80% across renal states (Table2).

**Figure:**
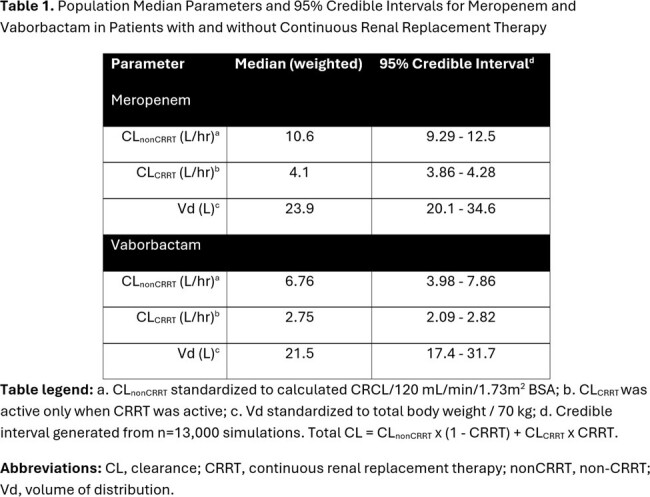

**Conclusion:**

CRRT patients had roughly 40% lower MV CL vs. non-CRRT patients. VAB had lower CL vs. MEM resulting in accumulation. Aggressive (e.g., 100% *f*T _>MIC_) MV targets may be challenging to attain vs. contemporary isolates while target attainment vs. susceptible isolates remains high.

**Figure:**
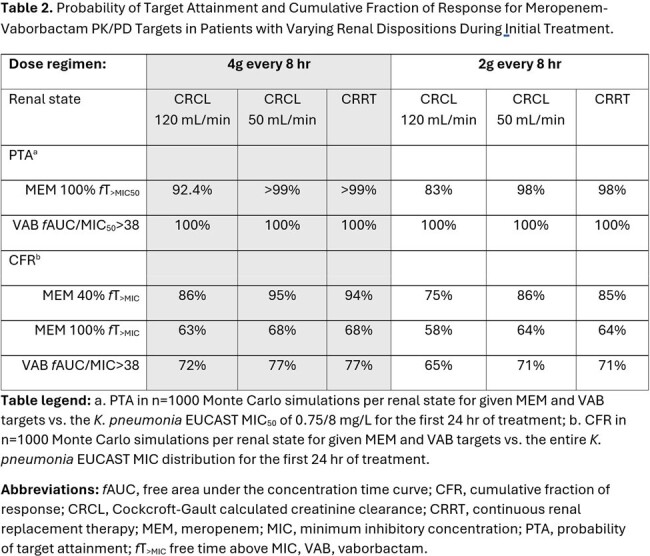

**Disclosures:**

**Erin K. McCreary, PharmD**, Abbvie: Advisor/Consultant|Basilea: Advisor/Consultant|Ciadara: Advisor/Consultant|Entasis: Advisor/Consultant|Ferring: Advisor/Consultant|GSK: Advisor/Consultant|GSK: Honoraria|Melinta: Advisor/Consultant|Merck: Advisor/Consultant|Pfizer: Honoraria|Shionogi: Advisor/Consultant|Shionogi: Honoraria **Brandon Smith, MD, PharmD**, Melinta Therapeutics: Advisor/Consultant|Shionogi, INC: Advisor/Consultant **Ryan K. Shields, PharmD, MS**, Allergan: Advisor/Consultant|Cidara: Advisor/Consultant|Entasis: Advisor/Consultant|GSK: Advisor/Consultant|Melinta: Advisor/Consultant|Melinta: Grant/Research Support|Menarini: Advisor/Consultant|Merck: Advisor/Consultant|Merck: Grant/Research Support|Pfizer: Advisor/Consultant|Roche: Grant/Research Support|Shionogi: Advisor/Consultant|Shionogi: Grant/Research Support|Utility: Advisor/Consultant|Venatorx: Advisor/Consultant|Venatorx: Grant/Research Support **Nathaniel J. Rhodes, PharmD MS**, Apothecademy, LLC: Advisor/Consultant

